# African Swine Fever: Vaccine Advancement and Major Gaps

**DOI:** 10.3390/microorganisms14030706

**Published:** 2026-03-21

**Authors:** Lihua Wang, Jishu Shi

**Affiliations:** 1 Center on Biologics Development and Evaluation, College of Veterinary Medicine, Kansas State University, Manhattan, KS 66506, USA; 2 Department of Anatomy and Physiology, College of Veterinary Medicine, Kansas State University, Manhattan, KS 66506, USA

**Keywords:** African swine fever, vaccine, advancement, gaps

## Abstract

African swine fever (ASF), a highly contagious and lethal viral disease caused by the African swine fever virus (ASFV), poses a severe threat to the global swine industry. Recent outbreaks across Asia, Europe, and the Caribbean are exacerbating the challenge. Current control measures rely mainly on early detection, culling and strict biosecurity practices, underscoring the urgent need for a safe and effective vaccine. Since the mid-1960s, diverse vaccine strategies, including inactivated, subunit, DNA/mRNA, vectored, and live attenuated virus (LAV) vaccines, have been explored. Inactivated vaccines have consistently failed to confer protection due to insufficient functional antigen presentation and weak cellular immune activation. Subunit vaccines targeting single or multiple ASFV antigens have also shown limited success, often failing to induce sterile or long-lasting immunity. Among these approaches, LAV vaccines have demonstrated the greatest promise in eliciting robust and durable immune responses. However, major knowledge gaps remain regarding ASFV biology, ASFV–host interactions, ASFV immune evasion mechanisms, protective and cross-protective immunity, stable cell lines for LAV production, virulence reversion of LAVs, and the lack of harmonized standards for evaluating vaccine safety and efficacy, all of which impede the development of safe and broadly effective ASF vaccines. This narrative review summarizes recent advances in ASF vaccine research and highlights the critical obstacles that must be overcome to achieve successful ASF vaccine development.

## 1. Overview of ASF and ASFV

African swine fever (ASF) is a highly contagious viral disease of swine that has posed a persistent threat to the global swine industry for over a century. In this narrative review, we focus on summarizing current progress and remaining challenges in the development of ASF vaccines. Relevant literature was identified through searches of major scientific databases, including PubMed, Web of Science, and Google Scholar, using combinations of keywords such as African swine fever virus (ASFV), ASFV vaccine, live attenuated vaccine, subunit vaccine, viral vector vaccine, and ASF vaccine development. Peer-reviewed articles published primarily within the past decade were prioritized to capture recent advances, while earlier studies were also included when they provided critical foundational insights into ASFV immunology, pathogenesis, and vaccine research. Publications were selected based on their relevance to ASF vaccine strategies, immunological mechanisms, safety considerations, and existing gaps that limit vaccine development. The collected studies were synthesized, together with the author’s perspectives, to provide an overview of current vaccine approaches and to highlight major knowledge gaps and future research directions.

ASF was first identified in the early 1900s in present-day Kenya by veterinary pathologist Robert Eustace Montgomery, who termed it “East African swine fever” [1]. Early outbreaks were linked to contact between domestic pigs and asymptomatic warthog reservoirs. ASF subsequently spread throughout sub-Saharan Africa and later reached Indian Ocean islands, including Madagascar in 1998 and Mauritius in 2007 [2,3]. In 1957, contaminated pork waste from Africa introduced ASF to Portugal [4], marking its entry into Europe. The disease’s global footprint expanded in the mid-20th century, with outbreaks in Brazil (1978) and multiple European and Caribbean nations, including France (1964), Italy (1967), Malta (1978), Belgium (1985), and the Netherlands (1986) [5].

**Figure 1:**
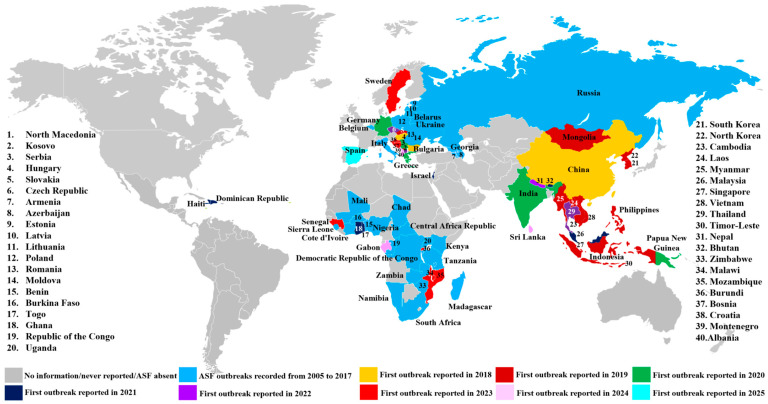
**Global distribution of ASF, 2005–2025**. This map is based on data from Situation reports for African swine fever of WOAH (https://www.woah.org/en/disease/african-swine-fever#ui-id-2, accessed on 26 January 2026) and Global Disease Monitoring Reports (https://www.swinehealth.org/global-disease-surveillance-reports, accessed on 26 January 2026). The names of countries with ASF are given on the map. Countries with continuing ASF outbreaks were labeled with the year when the first outbreak was reported since 2005.

A significant resurgence began in continental Europe in 2007, originating in Georgia [6] and spreading to Russia, Armenia, Azerbaijan, and Ukraine [7,8,9,10], affecting domestic pigs and wild boar populations. Between 2014 and 2018, ASF re-emerged in the European Union, impacting both domestic pigs and wild boars in Lithuania, Latvia, Poland, Estonia, Hungary, the Czech Republic, and Romania [11,12,13,14,15]. Recent expansions include outbreaks in Germany, Sweden, Italy, Bosnia, Herzegovina, Greece, and Croatia [16,17], again involving both wild and domestic *Suidae*. Despite years of impact in Europe, ASF’s arrival in China in 2018 triggered a global crisis, mainly affecting domestic pigs, with subsequent dissemination to other Asian countries, including Mongolia, Cambodia, India, Laos, Vietnam, the Philippines, North and South Korea, Myanmar, Timor-Leste, and Papua New Guinea, resulted in a dramatic halving of the regional swine population [18,19]. In 2021, ASF re-emerged in the Western Hemisphere on the Caribbean Island of Hispaniola (Dominican Republic and Haiti) after nearly 40 years [20,21], primarily in domestic pigs. Since 2005, ASF has spread across numerous countries on all inhabited continents except Antarctica (Figure 1), highlighting the immense challenge of controlling this persistent and highly contagious disease.

**Figure 2:**
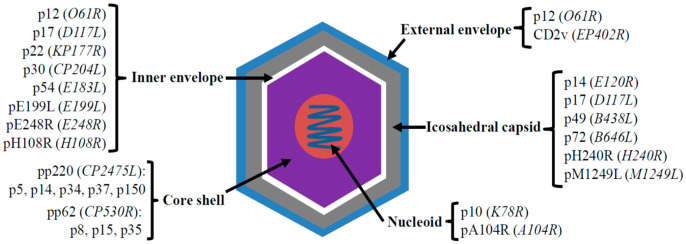
**Schematic of the ASFV virion, illustrating its layers and major structural proteins.** Genes encoding each protein are shown in *italics* within parentheses.

The causative agent of ASF is the ASFV, a large, enveloped, double-stranded DNA virus with predominantly cytoplasmic replication. Notably, ASFV is the only member of the *Asfarviridae* family and *Asfivirus* genus, and the only DNA virus known to be transmitted by arthropod vectors. Its genome, spanning 170 to 193 kilobase pairs, encodes approximately 150 to 200 proteins, with minor variations observed among different isolates [22,23,24]. ASFV virions are icosahedral particles, ranging from 170 to 300 nanometers in diameter, characterized by a complex structure of concentric layers: an external envelope, icosahedral capsid, inner envelope, core shell, and central nucleoid (Figure 2).

ASFV primarily targets macrophages, entering cells via dynamin- and clathrin-mediated endocytosis (CME) and micropinocytosis [25]. Although no specific receptors for ASFV have been identified, macrophage receptors such as CD163, CD45, and MHC II are assumed to play a role in viral entry [26]. ASFV initiates viral gene transcription immediately post-infection, utilizing its own encoded DNA replication enzymes (topoisomerase, helicases, polymerase, ligase, and binding protein), independent of host cellular machinery. Viral genome replication occurs in perinuclear factories through a complex, multi-stage process [27,28]. Virion assembly takes place on modified endoplasmic reticulum membranes, with mature virions budding from the plasma membrane [29]. ASFV manipulates host cell processes, including apoptosis and immune responses, to facilitate its replication and spread [30].

Twenty-four ASFV genotypes (I-XXIV), classified based on variations in the *B646L* gene (encoding p72), have been identified [31]. While all genotypes circulate in Africa [32], genotype I was initially responsible for the global spread of ASFV to Europe, Russia, South America, and the Caribbean. Genotype II ASFV was responsible for the re-emergence (since 2007) of ASFV in the Caucasus region and its subsequent appearance in the rest of the European and Asian countries. Recent studies have identified recombinant strains of genotypes I and II in Asia and Russia [33,34,35], posing significant new challenges for ASF control and vaccine development.

## 2. ASF Vaccine Development

Since the mid-1960s, a wide range of vaccine platforms, including inactivated, subunit, DNA/mRNA, vectored, and live attenuated virus (LAV) vaccines, have been explored for ASF. Inactivated vaccines, while exhibiting favorable safety profiles, have consistently shown poor efficacy, largely due to inadequate functional antigen presentation, epitope damage during inactivation, weak cellular immune activation, and the limited effectiveness of conventional adjuvants [36,37]. Subunit, DNA, and vectored vaccines targeting selected viral proteins are capable of eliciting measurable immune responses; however, they have frequently failed to confer sterile or durable protection. This limitation reflects the inherent challenges of rational protective immunogen selection arising from the structural and antigenic complexity of ASFV, as well as an incomplete understanding of protective immune mechanisms [38,39]. In contrast, LAVs have shown the greatest promise by providing enhanced immune protection through reduced virulence while preserving robust immunogenicity. Nevertheless, LAV development remains constrained by several challenges, including limited cross-protection among diverse ASFV genotypes, obstacles to large-scale manufacturing, concerns regarding genetic stability and safety for piglets and sows, and the need for reliable diagnostics to differentiate infected from vaccinated animals (DIVA). To date, only two commercial LAV vaccines (AVAC ASF Live and NAVET-ASFVAC) have been approved for controlled field use in Vietnam, and no other commercially approved ASF vaccines are available worldwide [40,41,42]. This review summarizes recent progress in the development of subunit, vectored, DNA/mRNA, and LAV vaccine platforms.

### 2.1. Protective Antigen-Based ASF Vaccines: Subunit, Vectored, and DNA/mRNA

Subunit vaccines remain one of the most intensively investigated strategies for ASF vaccine development. By focusing on defined viral proteins (antigens), they offer enhanced safety and compatibility with DIVA approaches. Initial investigations focused on key structural proteins, including p22, p30, p54, p72, and CD2v. Among these, the hemagglutinin (HA) protein CD2v was the first to be studied. Pigs (n = 3) immunized with baculovirus-expressed CD2v survived challenge with the homologous *E75* strain (genotype I); however, two pigs remained viremic for at least 28 days, with viral titers ranging from 10^5.35^ to 10^6.02^ TCID_50_ (50% tissue culture infectious dose) [43]. Subsequent research focused on p30 and p54, which are involved in virus attachment and internalization that can induce neutralizing antibodies. Although individual immunization proved insufficient [44], pigs (n = 2) immunized with a p54/p30 chimera survived *E75* challenge but experienced approximately two weeks of clinical signs (fever, recumbency, and anorexia) and viremia [45]. Additionally, a cocktail of p22, p30, p54, and p72 failed to protect pigs against challenge with the *Pr4* strain (genotype I) [46]. Researchers are expanding beyond classical antigens by screening the entire ASFV proteome to find novel epitopes and immunogenic proteins that might improve vaccine breadth and efficacy. Recently, a Chinese company reported a challenge trial of a multi-protein ASF subunit vaccine in pigs. According to the report, all 10,035 vaccinated pigs survived a challenge (oral spray) with the virulent genotype II ASFV strain (*HB19*), whereas 100% of the 970 unvaccinated control pigs succumbed to infection [47]. However, these findings have not yet been described in a peer-reviewed publication, and detailed information regarding the experimental design, immunological responses, and protective mechanisms is not publicly available. Therefore, the reported efficacy should be interpreted cautiously until independent studies and mechanistic data become available. In particular, it remains unclear whether antibodies against selected ASFV proteins alone would be sufficient to confer protective immunity, or whether additional immune mechanisms, such as T-cell-mediated responses, are required.

Various vectored platforms, including viral vectors like adenovirus, alphavirus, baculovirus, Modified Vaccinia Ankara (MVA), Newcastle disease virus (NDV), and pseudorabies virus (PRV), as well as microbial vectors such as bacteria and yeast, have been explored to deliver ASFV antigen-encoding DNA into host cells, aiming to stimulate both humoral and cell-mediated immune responses in pigs (Table 1). Among them, adenovirus-based vectors have been the most extensively studied, with variable efficacy reported. Pigs (n = 9) immunized with adenoviral combinations expressing multiple ASFV antigens (p30, p54, p72, pp62, pp220) achieved 55.5% survival following challenge with *Georgia 2007/1* strain (genotype II) [48,49,50]. An alternative adenoviral antigen set (p54, p72, pE199L, pEP153R, pF317L, pMGF505-5R) achieved 80% survival in pigs (n = 5) challenged with the *OUR T1988/1* strain (genotype I) [51]. Baculovirus-mediated expression of fused p30, p54, and CD2v antigens resulted in a 66.7% survival in pigs (n = 6) following challenge with the *E75* strain (genotype I) [52]. Additionally, a recombinant vaccinia virus strategy combining rTTV-D-A and rTTV-K-J, which together express eight ASFV genes (p17, p30, p49, p54, p72, CD2v, pB602L, pH240R) along with a synthetic T antigen composed of conserved T-cell epitopes, achieved 66.7% survival in pigs (n = 6) challenged with the *HLG/18* strain (genotype II) [53]. In contrast, although an NDV-based vector expressing p22, p72, pB602L, and pEP84R elicited robust humoral and cellular immune responses, it failed to protect pigs against challenge with the *ASFV-SY18* strain (genotype II) [54]. Immune responses induced by other vectored platforms, including PRV, bacterial, and yeast-based systems, also have been reported [55,56,57,58]. However, their protective efficacy in swine challenge models has not yet been evaluated (Table 1).

Based on the potential of inducing high levels of specific T cell responses, DNA vaccines have been explored as an alternative strategy for ASF vaccine (Table 1). DNA vaccines enhance immunogenicity by facilitating intracellular antigen expression and presentation via MHC-I molecules, a key process in activating CD8+ T cells. However, initial attempts using DNA constructs encoding the p54/p30 fusion protein failed to confer protective immunity in pigs [59,60]. Subsequent studies showed that a DNA vaccine combining the haemagglutinin extracellular domain with p54 and p30 antigens (sHA/p54/p30) elicited both cell-mediated and humoral immune responses, yet it did not provide protection against *E75* challenge [59]. Notably, the addition of ubiquitin to the sHA/p54/p30 fusion construct improved efficacy, resulting in 33% survival in pigs (n = 6) challenged with *E75* [60]. Moreover, pigs (n = 10) immunized with a DNA expression library containing multiple ASFV open reading frames (ORFs) linked to ubiquitin achieved 60% survival following *E75* challenge [61]. This survival of challenged pigs in these studies was associated with activation of ASFV-specific T cells, underscoring the critical role of cell-mediated immunity in controlling ASFV infection.

For mRNA-based approaches, a cocktail of six ASFV antigen-encoding mRNAs (*B602L*, *EP402R*, *EP153R*, *CP204L*, *E183L*, and *B646L*) formulated with lipid nanoparticles (LNPs) elicited robust humoral and cellular immune responses in both mice and pigs (Table 1) [62]. In addition, computational and bioinformatics-driven strategies have been employed to design multiepitope, pan-proteomic mRNA vaccine candidates with strong predicted immunogenicity in silico, providing a foundation for subsequent experimental validation [63]. Collectively, while DNA and mRNA platforms show promise in inducing both cellular and humoral immunity against ASFV, further optimization of multi-antigen and multi-epitope formulations, potentially combined with improved adjuvants and advanced delivery systems, will be necessary to achieve robust and durable protection against virulent ASFV challenge.

**Table 1:** Subunit, vectored, and DNA/mRNA vaccine candidates evaluated in pigs.

Vaccine Type		Antigen/Gene (*ASFV Source*)	Humoral Response		Cellular Response	Survival Rate (Challenge Strain)	Reference
			Specific Antibodies	Neutralizing Antibodies			
Subunit (protein)		CD2v (*E75CV*_1_)	Yes	NT	NT	100%, n = 3/3 (*E75*)	[43]
		p30 + p54 (*E70*)	Yes	Yes	NT	50%, n = 3/6 (*E75*)	[44]
		Chimeric p30/p54 (*E75*)	Yes	Yes	NT	100%, n = 2/2 (*E75*)	[45]
		p22 + p30 + p54 + p72 (*Pr4*)	Yes	Yes	NT	0%, n = 0/6 (*Pr4*)	[46]
Adenovirus-vectored		CP204L + E183L + CP530R + B646L (*Georgia 2007/1*)	Yes	NT	Yes	NT	[48,49,50]
		A151R + B119L + B602L + EP402RΔPRR + B438R + K205R-A104R (*Georgia 2007/1*)	Yes	NT	Yes	NT	[49]
		B646L + CP204L + CP2475L + CP530R + E183L (*Georgia 2007/1*)	Yes	NT	Yes	55.5%, n = 5/9 (*Georgia 2007/1*)	[50]
		B602L + E183L + E199L + EP153R + F317L + MGF505-5R (*OUR/T88/3* and *Benin 1997/1*)	Yes	NT	Yes	80%, n = 4/5 (*OUR T1988/1*)	[51]
Baculovirus-vectored		Fusion sHA/E183L/CP204L (*E75*)	No	NT	Yes	66.7%, n = 4/6 (*E75*)	[52]
Recombinant vaccinia-vectored		EP402R + B646L + B602L + D117L + H240R + B438L + E183L + CP204L (*Pig/HLJ/2018*)	Yes	NT	Yes	66.7%, n = 4/6 (*HLJ/18*)	[53]
Newcastle disease virus-vectored		B602L + EP84R + KP177R + B646L (*ASFV-SY18*)	Yes	Yes	Yes	0%, n = 0/4 (*ASFV-SY18*)	[54]
Pseudorabies virus-vectored		B646L + B602L (*PIG/HLJ/2018*)	Yes	NT	NT	NT	[55]
*Lactococcus lactis*-vectored		CP204L + E183L + B646L (*BA71V and VNUA Hanoi-ASF9 and Spencer*)	Yes	NT	Yes	NT	[56]
*Salmonella enterica* subsp. *enterica serovar* Typhimurium-vectored		B119L + EP402R + EP153R + O61 + E183L + B464L (*Genotype II ASFV*)	Yes	NT	Yes	NT	[57]
*Saccharomyces cerevisiae*-vectored		KP177R + E183L + E199L + CP204L + E248R + EP402R + B602L + B646L (*Pig/HLJ/2018*)	Yes	NT	Yes	NT	[58]
DNA		CP204L/E183Lfusion (*E75*)	No	NT	No	0%, n = 0/4 (*E75*)	[59]
		sHA + CP204L + E183L (*E75*)	Yes	No	Yes	0%, n = 0/6 (*E75*)	[60]
		Ub + sHA + CP204L + E183L (*E75*)	Yes	No	Yes	33%, n = 2/6 (*E75*)	[60]
		Library spanning about 76% of the genome (*Ba71V*)	No	NT	Yes	60%, n = 6/10 (*E75*)	[61]
mRNA		B602L + EP402R + EP153R + CP204L + E183L + B646L (*Pig/HLJ/2018*)	Yes	NT	Yes	NT	[62]
Prime-boost	rAdenovirus, rMVA	B602L + B646L + CP204L + E183L + E199L + EP153R + F317L + MGF505-5R (*OUR/T88/3 and Benin 1997/1*)	Yes	NT	Yes	100%, n = 6/6 (*OUR/T88/1*)	[64]
	DNA, rVACV	47 ASFV antigen genes (*Georgia 2007/1*)	Yes	No	Yes	0%, n = 0/6 (*Georgia 2007/1*)	[65]
	DNA, Protein	DNA (EP402R + B646L + CP204L +/− D 117L), protein (p15, p35, p54, +/− p17) (*Ba71V and E70*)	Yes	No	Yes	0%, n = 0/5 (*Armenia 2007*)	[66]
	Protein/rMVA, Protein/rMVA	rMVA: B646L + EP153R + EP402R; Protein: p72 + CD2v + C-type lectin (*Georgia 2007/1*)	Yes	NT	Yes	NT	[67]
	Alphavirus, LAV	CP204L (*Ba71V*) + *OURT88/3*	Yes	Yes	NT	NT	[68]
	DNA, LAV	M448R + MGF505-7R (*Georgia2007/1*) + *BA71∆CD2*	Yes	NT	Yes	60%, n = 3/5 (*Georgia2007/1*)	[69]

Note: “sHA”, extracellular/soluble domain of hemagglutinin (CD2v); “rMVA”, recombinant Modified Vaccinia Ankara vector which harbors the ASFV antigen genes; “rVACV”, recombinant vaccinia virus; “Ub”, cellular ubiquitin; “NT”, not tested.

### 2.2. Prime-Boost Vaccination with Different Vaccine Platforms

Prime-boost strategies, which employ two or more distinct vaccine platforms to enhance both humoral and cellular immune responses, have been investigated (Table 1). In one study, pigs (n = 6) primed with replication-deficient human adenovirus type 5 (rAd) vectored eight ASFV genes (*B602L*, *B646L*, *CP204L*, *E183L*, *E199L*, *EP153R*, *F317L*, and *MGF505-5R*) and boosted with Modified Vaccinia Ankara (MVA) expressing the same genes with the exception of *MGF505-5R* survived challenge with the virulent genotype I *OUR T1988/1* strain [64]. However, all pigs developed pyrexia (with temperatures in some cases above 40.5 °C and reaching in many cases values above 41 °C), accompanied by clinical signs and viremia peaking at 5–6 days post-challenge; viremia persisted until the end of the study (20 days post-challenge). In contrast, when evaluated against highly virulent genotype II strains, prime-boost approaches combining DNA vaccines with recombinant poxvirus-vectored or subunit vaccines have largely failed to achieve effective protection. For instance, a heterologous prime-boost strategy in which pigs were primed with 47 plasmid DNA constructs and boosted with 47 recombinant vaccinia viruses did not protect vaccinated animals, although reductions in viral loads were observed in blood and selected tissues [65]. Similarly, a vaccine cocktail comprising p15, p17, p30, p54, p72, and CD2v delivered using a DNA prime-protein boost regimen induced antigen-specific antibody responses to p15, p35, and p54. But antigen-specific T-cell responses were limited, and vaccinated pigs (n = 5) were not protected following lethal challenge with the *Armenia 2007* strain [66]. Additional prime-boost approaches have also been investigated. Prime with MVA vector expressing individual p72, CD2v, and C-type lectin antigens, followed by a boost with the corresponding mammalian cell-expressed proteins, induced ASFV-specific antibody and T-cell responses in pigs [67]. An alphavirus vector delivering p30, used in combination with an attenuated *OURT88/3* strain, elicited a strong anti-p30 antibody response and enhanced recognition of the p30 epitope spanning amino acids 61–110 [68]. Moreover, priming pigs (n = 5) with DNA plasmids encoding pM488R and pMGF505-7R, followed by boosting with the LAV *BA71ΔCD2*, resulted in 60% survival against a lethal challenge with the *Georgia2007/1* strain [69]. Collectively, these findings underscore both the potential and the limitations of prime-boost vaccination strategies. To improve efficacy, well-controlled studies in pigs are needed that carefully optimize the timing and sequence of prime and boost immunizations, evaluate combinations of multiple protective antigens, and compare different vaccine platforms, including subunit, viral vector, and live attenuated vaccines. Additionally, testing alternative delivery routes (e.g., intramuscular, oral, or intranasal) may enhance immune responses. These targeted approaches will help clarify the protective potential of prime-boost strategies and guide the development of vaccines capable of conferring robust and broad protection against virulent ASFV strains.

### 2.3. ASF Live Attenuated Virus (LAV) Vaccine

LAV vaccines are created by diminishing the pathogenicity of virulent virus strains while maintaining their ability to replicate and induce a robust immune response. Historically, this attenuation was achieved through physical methods such as serial passage in non-natural hosts or cell cultures, a process that forces the virus to adapt to a new environment, thereby losing its virulence in the original host (e.g., Oral Polio Vaccine). Modern vaccinology also employs genetic modification (e.g., Rotavirus vaccines) to achieve attenuation. For ASFV, LAVs are categorized into three types: naturally attenuated strains, subculture attenuated strains, and genetically manipulated attenuated strains.

Naturally occurring low-virulence mutants of ASFV, isolated from soft ticks, chronically infected domestic pigs, and wild boars, do not cause mortality in domestic pigs and provide immunological protection. Several such isolates have been identified and evaluated as potential vaccine candidates. Among these, *NH/P68* (genotype I), *OURT88/3* (genotype I), and *Lv17/WB/Rie1* (genotype II) are the most studied. *NH/P68*, a low-virulence strain isolated from a domestic pig exhibiting chronic ASF symptoms, and *OURT88/3*, isolated from a soft tick on a farm, are non-lethal and induce asymptomatic or chronic ASF lesions in domestic pigs. Notably, these viruses are non-hemadsorbing (non-HAD) [70,71]. *Lv17/WB/Rie1*, another non-HAD attenuated ASFV, was isolated from the serum of a hunted wild boar in Latvia. Experimental studies have shown that *Lv17/WB/Rie1* induces strong antibody responses, and pigs (n = 2) survived challenge with virulent ASFV genotype II isolates (e.g., *Armenia/07*). When administered orally to wild boar, 11 of 12 vaccinated and in-contact animals (exposed through contact) survived virulent challenge with *Armenia/07* [72,73]. Additionally, ASFV *DR21*, identified in the Dominican Republic, shows a 50% survival rate upon oronasal (ON) inoculation, with surviving animals exhibiting a mild form of the disease [21]. Despite their attenuated phenotype, these naturally occurring ASFV isolates can still establish persistent or chronic infections in domestic pigs, raising important safety concerns for their use as vaccines.

Early research in the 1960s explored adapting and attenuating ASFV through passaging in primary cell cultures, including bone marrow (BM), buffy coat (BC), pig leukocyte (PL), primary pig kidney cells (PPKs), primary bone marrow cells (PBMCs), and porcine alveolar macrophages (porcine AMs) [74,75]. However, the time-consuming, risk of contamination, and expensive nature of obtaining primary cells, along with ethical considerations, hampered large-scale production and research efforts. To address these limitations, researchers have adapted ASFV strains to continuous cell lines, including monkey-derived cell lines (e.g., COS-1, CV1, MA104), porcine-derived cell lines (e.g., CAS-01, IPAM, PIPEC, IPKM, PPK-66b, 3D4/21, A4C2/9K, ZMAC-4) [74,75,76,77,78,79]. This transition to continuous cell lines has significantly streamlined ASFV research and vaccine development. Following the ASF outbreak in 2018 in Asia, numerous cell-adapted LAVs have been developed globally, such as *ASFV-G/VP110*, *ASFV-MEC-01*, *Congo-a KK262*, *VNUA-ASFVLAVL2*, and *VNUA-ASFVLAVL3* [76,77,78,79,80,81,82,83,84]. Those LAVs can elicit robust humoral and cellular immune responses in pigs, showed promising as a LAV vaccine against homologous virulent virus challenge.

Advances in genome editing technologies and ASFV genomics have enabled the creation of gene-deleted LAV vaccine candidates. Several research groups have developed ASF LAVs through the deletion of genes associated with virulence, which elicit robust immunity against homologous strains. Examples include single-gene deletion LAVs (*ASFV-G-ΔI177L*, *ASFV-G-ΔA137R*, and *SY18ΔI226R*) and multiple-gene deletion LAVs (*HLJ/18-7GD*, *ASFV-G-ΔMGF*, *ASFV-GΔ9GL/ΔCD2v*, and *ASFV-G-ΔI177LΔLVR*) [41,85,86,87,88,89,90,91]. Currently, two LAVs, AVAC ASF Live and NAVET-ASFVAC, have received commercial approval in Vietnam. AVAC ASF Live is based on the *ASFV-G-ΔMGF* strain with six *MGF* gene deletions, while NAVET-ASFVAC is based on the *ASFV-G-ΔI177L* strain, developed by partially deleting the *I177L* gene [40,41,42,89,91].

However, the development of ideal LAV vaccines that meet the demands for widespread commercial use still faces challenges. Field application of these vaccines raises significant concerns, particularly regarding the potential for reversion to virulence. Reversion may occur through spontaneous point mutations, recombination with field strains, or genome rearrangements, which could restore pathogenicity. For example, *ASFV-G-ΔI177L* has been reported to severely impair reproductive performance of pregnant sows and is genetically unstable, with a high risk of reverting to virulence. Upon inoculation of two pregnant sows with ASFV-G-ΔI177L, one developed moderate ASF-related clinical signs. In terms of reproductive performance, 43% of the offspring were born dead and the live-born piglets developed ASF-specific clinical signs, became viremic, and only 17% survived until the end of study. During passaging in pigs, ASFV-G-ΔI177L reverted to virulence with severe ASF-specific clinical signs at passages 3 and 4, associated with increased viremia. Whole genome sequencing identified C257L mutations as a potential driver of increased replication fitness and virulence [92]. Additional concerns include the availability of stable cell lines for large-scale LAV production, variability in vaccine safety and efficacy across different pig ages, potential reversion to virulence, vaccine virus shedding, risk of virulence for other species or other *Suidae*, the ability to differentiate infected from vaccinated animals (DIVA), and cross-protection against diverse ASFV strains, particularly emerging recombinant variants [38,81,82,93,94].

## 3. Current Challenges in ASF Vaccine Development

Developing an effective vaccine against ASF has proven exceptionally challenging. Current ASF vaccine development efforts, employing various strategies, face various limitations (Table 2).

Major gaps persist in ASF vaccine development, especially:

(i) Incomplete understanding of protective immunity

Although both humoral and cellular immune responses contribute to protection, their relative roles remain poorly defined. Recent studies suggest that non-neutralizing antibodies can mediate protection through Fc-dependent effector mechanisms, including antibody-dependent cellular cytotoxicity (ADCC) and complement activation [95]. Additionally, several ASFV-specific T-cell epitopes have been identified that stimulate strong CD8^+^ and CD4^+^ T-cell responses, indicating that cellular immunity plays a critical role in controlling infection [69,96,97]. These findings underscore the need for vaccines that elicit a coordinated humoral and cellular response, a consideration critical for both subunit and LAV vaccine design.

(ii) Limited knowledge of viral immune evasion mechanisms

ASFV rapidly disrupts host immune responses during the earliest stages of infection, causing rapid depletion of immune cells. However, the mechanisms underlying this immune suppression remain incompletely understood. ASFV inhibits interferon (IFN-I, IFN-II, and IFN-III) responses, impairs NF-kB/NFAT and cGAS-STING pathways, modulates apoptosis, and suppresses T-cell activation [98]. These evasion mechanisms likely contribute to the limited efficacy of vaccines, as they can blunt the induction of protective humoral and cellular responses, underscoring the need for vaccine strategies that overcome or bypass viral immune suppression.

(iii) Uncertainty in antigen prioritization

Although numerous structural and non-structural ASFV proteins (e.g., p30, p54, p72, CD2v) have been evaluated as vaccine antigens, no single antigen or antigen combination has consistently conferred robust protection. Limited knowledge of immunodominant versus protective epitopes, together with genotype- and strain-specific antigenic variation, restricts the rational design of subunit, vectored, and DNA/mRNA vaccines.

(iv) Viral diversity, emergence of recombinant variants, and gaps in cross-protection

ASFV exhibits high genetic diversity and frequent recombination, particularly between genotype I and II viruses, giving rise to novel variants with potentially altered virulence and antigenicity. The durability of vaccine-induced immunity and the mechanisms underlying cross-protection among diverse ASFV genotypes remain poorly understood. These limitations pose major challenges for designing vaccines that are broadly protective across circulating and emerging ASFV strains.

(v) Safety and DIVA concerns with LAV vaccines

Despite their superior efficacy, LAVs raise persistent safety concerns. Key unresolved issues include the breadth of cross-protection, risk of virulence reversion, potential horizontal or vertical transmission, safety in pregnant sows, and the need for comprehensive safety datasets. In addition, antibodies induced by LAVs are difficult to distinguish from those elicited by natural infection, complicating disease surveillance and eradication programs.

(vi) Lack of harmonized standards for vaccine evaluation

The absence of internationally harmonized standards for assessing vaccine purity, potency, safety, and efficacy has hindered ASF vaccine research, regulatory approval, and market authorization. The adoption of WOAH standards in May 2025 represents a significant step forward [99]. Further detailed guidance, such as standardized protocols for field evaluation and post-vaccination monitoring, is currently under development [100]. Alignment with these standards is strongly encouraged for vaccine researchers and manufacturers to ensure regulatory compliance and the safe deployment of ASF vaccines. Beyond technical standardization, a multilateral collaborative architecture is the only viable paradigm for the global control and eventual eradication of ASF. Within an increasingly interconnected trade environment in the current world, international cooperation must be intensified to facilitate real-time data sharing and the synchronization of vaccination and movement controls. Such global synergy is a fundamental prerequisite for safeguarding international food security and the socio-economic stability of producers worldwide.

Collectively, these gaps substantially constrain the rational design, optimization, evaluation, and application of ASF vaccines. Addressing these fundamental knowledge gaps is essential for advancing next-generation ASF vaccine platforms that are safe, effective, and broadly protective. A coordinated and collaborative approach will be critical for the successful control and eventual eradication of ASF. Governments and the international swine industry should work together to establish, harmonize, and strictly implement standardized criteria and biosecurity measures worldwide, thereby safeguarding global food security and the socio-economic stability of pork producers.

## 4. Future Perspectives

Progress in ASF vaccine development hinges on sustained investment and a deeper mechanistic understanding of ASFV biology and host–virus interactions. Systematic functional characterization of ASFV proteins, particularly those involved in immune evasion, immunosuppression, and virulence, is central to rational vaccine design. In this context, comprehensive proteome-wide screening combined with modern virological and immunological approaches will help advance this effort. These include high-throughput CRISPR screens to identify host factors essential for ASFV replication, reverse genetics integrated with AI-assisted analysis, and multi-omics and systems immunology strategies to identify protective antigens and define correlates of immunity. In addition, standardized challenge models using diverse recombinant ASFV strains will be important for evaluating cross-protection and establishing robust correlates of protection.

Greater clarity regarding humoral and cellular immune mechanisms, as well as the pathways by which ASFV subverts innate immunity, is urgently needed. In parallel, understanding the mechanisms of virulence reversion and ensuring robust DIVA strategies must remain central considerations, particularly for LAV vaccines. Recent efforts have focused on developing marker vaccines through targeted deletion of immunogenic ASFV genes, enabling the design of companion diagnostic assays that detect antibodies against the deleted proteins. In parallel, PCR-based assays targeting the deleted genomic regions are also being explored to support DIVA-compatible surveillance. Continued development of such marker vaccines and companion diagnostics will be critical for enabling safe deployment of LAVs while maintaining effective disease monitoring and control programs. There is no single “ideal” ASF vaccine; rather, different vaccine platforms may serve complementary roles depending on epidemiological context. LAV vaccines may provide rapid outbreak control in high-risk settings, whereas protective antigen-based ASF vaccines (subunit, vectored, and DNA/mRNA) may support long-term disease management and eradication. Strategic, context-dependent deployment combined with continued optimization of LAV and protective antigen-based vaccines may ultimately bridge the gap between efficacy and safety.

Finally, experiences from Asia underscore the necessity of stringent biosafety measures, comprehensive surveillance, and rigorous regulatory oversight. Ensuring vaccine quality through standardized evaluation, long-term field monitoring, and independent safety assessment will be essential for building confidence and enabling responsible global deployment. Although challenges remain, continued scientific advances suggest that safe and effective ASF vaccines are an achievable goal in the near future.

## Figures and Tables

**Table 2:** Current strategies, limitations, and development status of ASF vaccines.

Vaccine Type	Mechanism to Induce Immune Responses	Advantages	Limitations	Status
**Inactivated**	Killed virus with adjuvants	Safe, no risk of disease from vaccine	Weak humoral and cellular responses, fail to produce long-lasting immune response and adequate protection, lack DIVA ability	Currently, not a focus
**Subunit**	ASFV protective antigen with adjuvants	Safe, targeted immune response, DIVA capability	Requires adjuvants, need to identify specific protective antigens, may require multiple doses	In development
**Viral vector**	ASFV protective antigen	Safe, targeted immune response, DIVA capability, single dose possible	Potential pre-existing immunity to vector, need to identify specific protective antigens	In development
**DNA**	ASFV protective antigen	Safe, targeted immune responses, DIVA capability	May require multiple doses, need to identify specific protective antigens	In development
**mRNA**	ASFV protective antigen	Safe, targeted immune responses, DIVA capability	Requires cold storage, need to identify specific protective antigens	In development
**Live attenuated**	Weaked virus	Strong immune responses (humoral and cellular) and superior efficacy, single lower dose possible	Need stable cell lines for large-scale production, risk of reversion to virulence, risk of recombination, lack DIVA ability, lack cross-protection	In use in restricted area

## Data Availability

No new data were created or analyzed in this study. Data sharing is not applicable to this article.
